# Timing Robustness in the Budding and Fission Yeast Cell Cycles

**DOI:** 10.1371/journal.pone.0008906

**Published:** 2010-02-01

**Authors:** Karan Mangla, David L. Dill, Mark A. Horowitz

**Affiliations:** 1 Department of Computer Science, Stanford University, Stanford, California, United States of America; 2 Department of Electrical Engineering, Stanford University, Stanford, California, United States of America; Mount Sinai School of Medicine, United States of America

## Abstract

Robustness of biological models has emerged as an important principle in systems biology. Many past analyses of Boolean models update all pending changes in signals simultaneously (i.e., synchronously), making it impossible to consider robustness to variations in timing that result from noise and different environmental conditions. We checked previously published mathematical models of the cell cycles of budding and fission yeast for robustness to timing variations by constructing Boolean models and analyzing them using model-checking software for the property of speed independence. Surprisingly, the models are nearly, but not totally, speed-independent. In some cases, examination of timing problems discovered in the analysis exposes apparent inaccuracies in the model. Biologically justified revisions to the model eliminate the timing problems. Furthermore, in silico random mutations in the regulatory interactions of a speed-independent Boolean model are shown to be unlikely to preserve speed independence, even in models that are otherwise functional, providing evidence for selection pressure to maintain timing robustness. Multiple cell cycle models exhibit strong robustness to timing variation, apparently due to evolutionary pressure. Thus, timing robustness can be a basis for generating testable hypotheses and can focus attention on aspects of a model that may need refinement.

## Introduction

Since reaction rates vary widely both because of the stochastic nature of interactions at low copy numbers and from diverse and dynamically changing environmental conditions, cells that grow and divide robustly in the face of these variations can be expected to have a competitive advantage over less robust cells. The principle of robustness to variation in reaction rates can be exploited to check the accuracy of models, and to narrow the range of possibilities when incomplete biological knowledge gives rise to multiple plausible models.

There are a variety of approaches available to define and evaluate robustness to noise, variation of parameters, and environmental conditions. One approach is to model the dynamics of the system with differential equations, and numerically integrate many times for various values of parameters to compute trajectories of the various signals in the system. However, this approach is problematic because there is a lack of detailed knowledge of quantitative reaction kinetics for most of the reactions in a cell, and because only a small fraction of the space of possible parameters can be examined.

Given the lack of quantitative information about reaction kinetics, a reasonable approach is to use Boolean models, which represent concentrations of proteins with a few discrete qualitative levels (strictly interpreted, the term “Boolean” implies variables can only take two values, but we use it in the more general sense that allows more than two discrete levels variables). Variations in reaction rates are reflected in Boolean models as variations in the *timing* of transitions between qualitative levels of reactants and products. The ability of a cell to maintain its function in the presence of timing perturbations is called *timing robustness*.

Since the 1960's [1], the dynamics of Boolean models of biological control have been based on the *synchronous update rule*: At each point in time, all variables that are logically able to change are updated simultaneously at the next time step. This approach has yielded many interesting results, but it allows no variation in timing, and consequently makes examination of timing robustness impossible. There are many ways to introduce varying amounts of freedom in the timing of reactions. A model that makes weaker assumptions about timing is more conservative, in that it is more likely to violate a given specification than a model with stronger timing assumptions. For example, the model could allow variables to change in one or two steps after they are enabled [2], [3], or variables could change within a continuous but bounded window, as can be specified with timed automata [4], or reactions could be classified as “fast” and “slow” reactions, with “fast” reactions occurring (effectively) infinitely faster than slow reactions [5], [6]. One of the most conservative models, which we call *fully asynchronous*, assumes nothing about the timing of reactions. The delay of a transition could be any finite time. A system that has a desired property in a fully asynchronous model is *speed-independent*. Speed independence implies timing robustness in most other plausible models. In particular, if the cell cycle works correctly in the fully asynchronous model, it also works in a synchronous model.

This paper explores the timing robustness of several Boolean models of the cell cycles of budding yeast (*S. Cerevisiae*) and fission yeast (*S. Pombe*). The Boolean model of budding yeast was published previously, and we derived the fission yeast models from published differential equations models. To test for timing robustness, our approach was to begin with a fully asynchronous model in each case, examine the cases where the models fail to be robust, and either change the model or introduce additional constraints on timing (making the model less-than-fully asynchronous) as necessary. Surprisingly, it was not necessary to weaken the assumption of full asynchrony. With changes that are either justified from the literature or at least as plausible as the original model, the cell cycle models could be made fully speed-independent. These results indicate that (1) the cell cycle is highly timing-robust, and (2) analyzing models for timing robustness can generate testable predictions about the details of cell cycle control.

To check for speed independence, we analyze the behavior of cell cycle models using the *asynchronous update rule* [7], which allows at most one variable to change at a time. When multiple variables are enabled to change, one of them is chosen arbitrarily. The asynchronous update rule models a system in which the delays are assumed to be arbitrary – nothing is known about the delays except that they are finite. In such a model, the system behavior depends only on the order in which variables change values. The situation where two signals may transition in the “wrong order,” leading to a disruption of the normal function of the cell, is called a *hazard*. If there are no hazards (i.e., if the model satisfies the specified property for all possible orders of variable transitions, corresponding to all possible delays), the model is speed-independent.

The property that a cell successfully completes cell division for all possible delays was checked using the NuSMV model checker [8], which systematically explores all of the possible orders in which variables can change. When this property failed, the cause of the hazard was diagnosed and the biological literature re-examined to determine whether revisions to the model could be justified from biological evidence. In all cases, hazards could be eliminated by biologically plausible revisions, and in some cases, these revisions could be considered corrections to the biology in the model.

Previous work has noted that the synchronous update rules is unrealistic, and explored the effects of introducing random variation in the update times [9]–[11]. Unlike that work, our speed-independent model has no concept of probability; it answers whether *every* possible timing of reactions results in a successful cell cycle.

Others have used model checking and similar techniques to explore the state space of biological systems [3], [5], [12], [13]. In particular, we found previously that *Caulobacter Crescentus* was almost speed-independent [6] (in that work, it was necessary to add a small number of timing constraints to the Caulobacter model to eliminate all hazards). However, in previous work, timing robustness appears not to have been used to critique and improve Boolean models, nor has selection pressure for speed independence been demonstrated *in silico*.

## Results and Discussion

Hazards can be detected by the use of software tools, called *model checkers*, that check a specified logical property on every possible state of a Boolean model. Model checking can be used with a synchronous or asynchronous update rule. Model checking with asynchronous updating effectively checks the property for *all possible* delays by systematically exploring every possible order in which enabled signals can occur. The inputs to a model checker consist of a boolean model, described as a set of variables and rules that modify their values, an initial state, and a logical property to check.

### Timing Robustness of the Budding Yeast Cell Cycle

**Figure 1:**
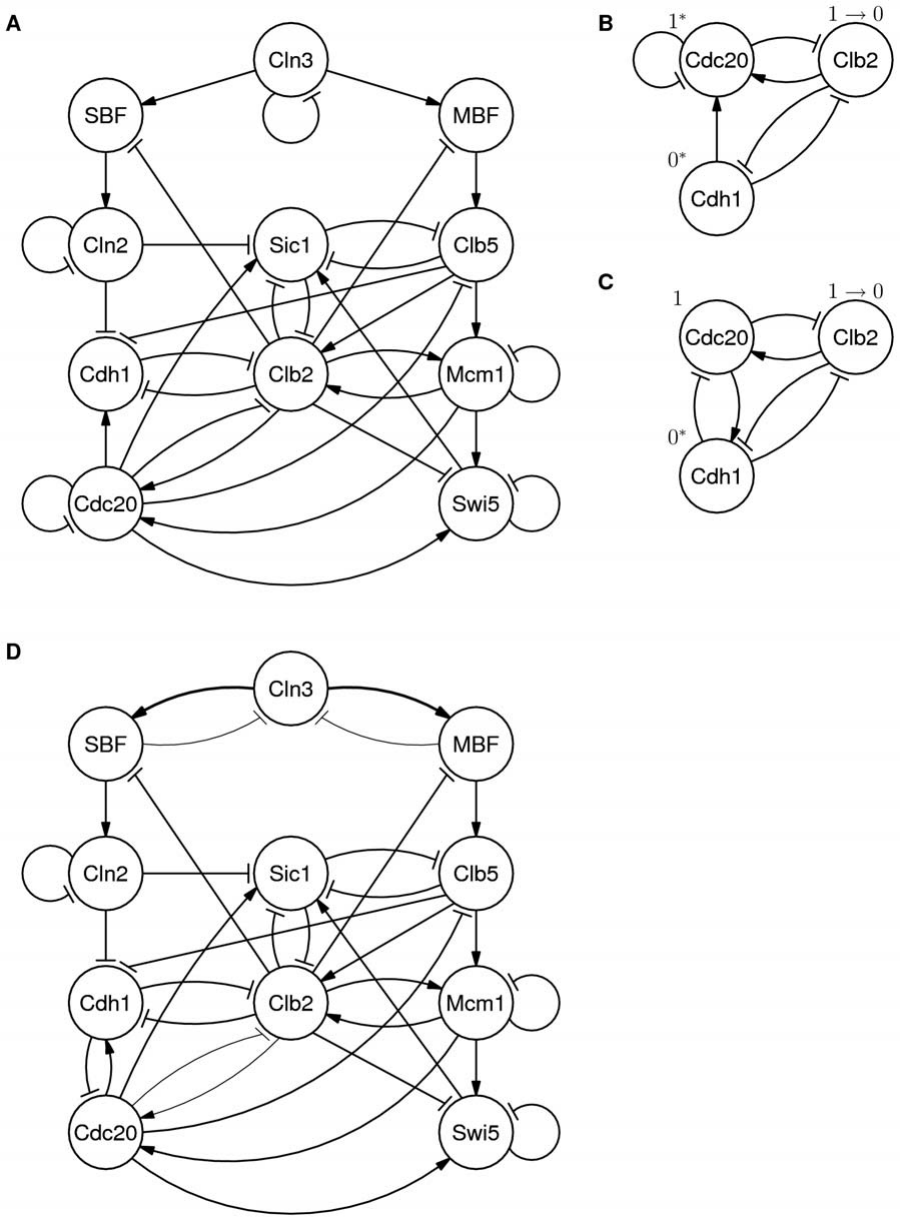
Budding yeast models. Nodes in the graph represent molecules, complexes, etc. Arrows with pointed heads represent activation, and arrows with bars indicate inhibition. Thin arrows represent a weight of 1/3, normal arrows represent a weight of 1 and thick arrows represent weight 3. (A) The model from Li, et al. [14]. (B) A subset of the model that highlights the first timing hazard. Nodes with values marked with * are enabled to change. If Cdc20 transitions from 1 to 0 before Cdh1 transitions from 0 to 1, Cdh1 will stay at 0, causing the cell cycle to arrest before it has returned to G1. (C) The hazard can be eliminated by replacing Cdc20 self-degradation with inhibition of Cdc20 by Cdh1, ensuring that Cdh1 transitions to 1 before Cdc20 transitions to 0. (D) The final speed-independent model for budding yeast.

A simplified Boolean model of the budding yeast cell cycle of (Figure 1A) was proposed and evaluated with a synchronous update rule by Li, et al. in 2004 [14]. In this model, nodes are labeled with protein names. At any given time, each node has a *level* representing a qualitative degree of activation of the protein. The activation state of a protein may represent that it is modified, forms a complex with other proteins, or is simply present so as to have an effect on other nodes in the system.

The originally published model had a node, Start, representing the conditions that initiate transition from the G1 to S phase of the cell cycle. We removed this node because it does not represent a real molecule. Instead, the initial state of our model is the same as G1, except that Cln3 is 1, representing that the cell cycle has just entered S. NuSMV was used to check the property that, from this state, the model inevitably leads back to G1, where it halts.

The first hazard is shown in Figure 1B. Cell cycle progression can reach a state where Cdh1 is enabled to change from 0 to 1 and Cdc20 is ready to change from 1 to 0. If Cdh1 changes first, the cell cycle proceeds normally and reaches G1, but, if Cdc20 changes first, Cdh1 stops trying to change, causing the cell cycle to halt before reaching G1.

The model can be revised to eliminate this hazard by replacing the self-inhibition of Cdc20 with inhibition of Cdc20 by Cdh1, as shown in Figure 1C, ensuring that Cdh1 transitions to 1 before Cdc20 transitions to 0. This change is supported by Huang, et al. [15], who show that APC-Cdh1 accelerates the degradation of APC-Cdc20 *in vivo*.

Continuing to analyze and revise successive models reveals a series of hazards, each of which can be resolved by small revisions until a fully hazard-free model is finally obtained. The second hazard occurs after a transition in Clb5 causes Clb2 to transition to 1, after which Cdc20 and Mcm1 are both enabled to transition to 1. If Cdc20 transitions first, Mcm1 can be disabled causing the cell to exit mitosis without Mcm1 ever going high, causing the cell cycle potentially to fail to enter G1.

According to the literature [16], [17], activation of Clb5 first causes Clb2 to be transcribed at a low level, which is sufficient to activate Clb2's own transcription factors, Mcm1/SFF (represented as Mcm1 in the model), which then cause Clb2 to be transcribed at a higher level.

To capture this understanding, the model was revised so that Clb2 has one of three possible values: 0, representing a negligible concentration of Clb2; 1, representing a low concentration; and 2, representing a high concentration. The modeling formalism of Li, et al. [14] was extended to permit three-valued signals (more details in Methods). The model was additionally modified so that Clb2 must be 2 before Cdc20 transitions to 1, eliminating the hazard by ensuring that Mcm1 transitions to 1 before Clb2 transitions to 2.

The revised model has one more hazard. When Cln3 becomes 1, it enables both MBF and SBF to transition from 0 to 1. But Cln3 inhibits itself, so it can return to 0 before either SBF or MBF changes, causing the cell cycle to arrest. The hazard can be eliminated by replacing self-degradation of Cln3 with inhibition by SBF and MBF, so that SBF and MBF both must change before Cln3 returns to 0. As observed in Orlando, et al. [18], MBF and SBF indirectly inhibit Cln3 by activating Yox1 and Yhp1.

With this revision, there are no more hazards. Upon entering the cell cycle from G1, the cell inevitably returns to G1 for every possible combination of delays at each of the nodes.

Another Boolean model of budding yeast by Irons appeared recently [19] that is more complete and introduces more molecular components. Interestingly this model includes most of the revisions discussed above, except for the inhibition of Cdc20 by Cdh1. Analysis of this model using NuSMV with the asynchronous update rule shows that it suffers from the same hazard involving Cdc20 and Cdh1 as the model of Li, et al. shown in Figure 1D. The Irons model has additional hazards involving the new components that were added beyond the Li *et al.* model, and it is unclear how to resolve them because they have not been extensively discussed in the biological literature. While a deeper study of hazards in the Irons model would be merited in the future, the fact that the model has the same issues as the simpler and more tractable models discussed above indicates that a simple model can reveal insights into issues that also occur in more complex models.

### Timing Robustness of the Fission Yeast Cell Cycle

The biology of fission yeast is less well documented than that of budding yeast, making it difficult to provide biological justifications for revisions based on hazards in the cell cycle. However, timing robustness can be used to compare models and suggest possible revisions.

We constructed three Boolean models based on previously published ODE models. In each case, we started with the “wiring diagram” presented in the ODE paper, adjusted the edge weights and made minor changes until the cell cycle worked properly under the synchronous update rule. The first model was based on a paper by Sveiczer, et al. in 2000 [20]. Analysis with NuSMV revealed many hazards involving a hypothetical protein, PP, that appeared in the model. Revisions to eliminate these hazards seemed pointless, since there is no biology to refer to. A Boolean model of fission yeast was published recently [21], also based on Sveiczer, et al., 2000 but that was similar to the model we constructed.

**Figure 2:**
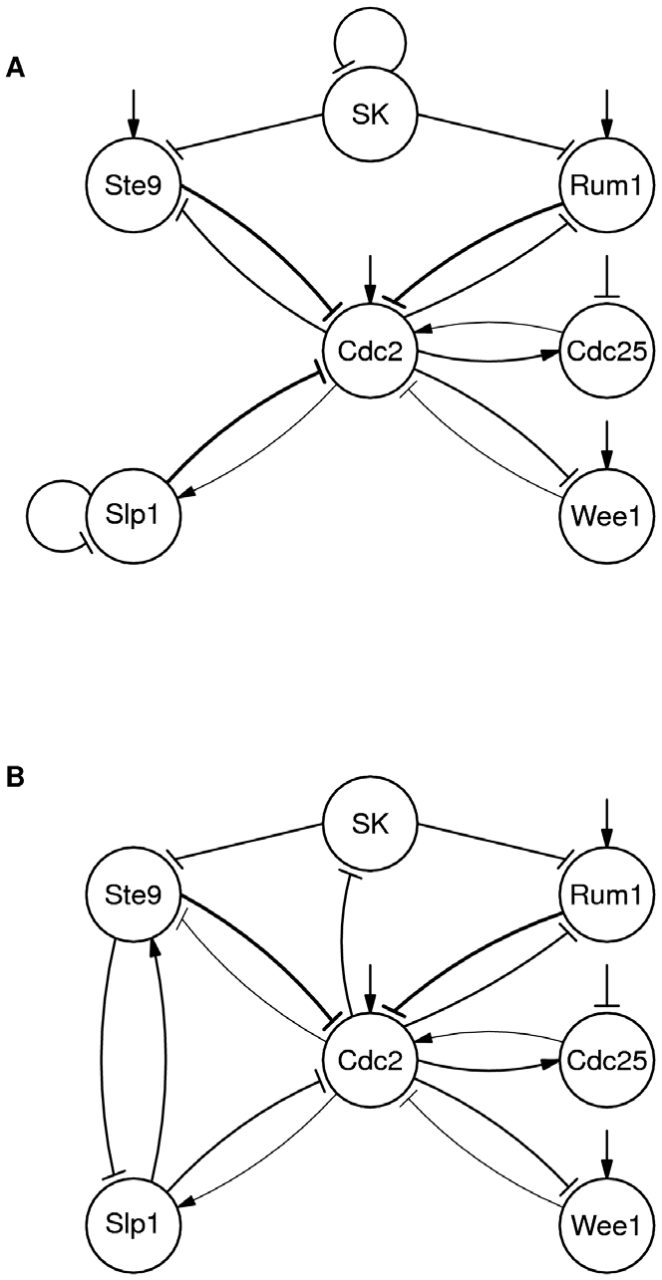
Fission yeast models. (A) Fission yeast model derived from Sveiczer, et al, 2004 [23]. (B) Revised speed-independent model.

We created two other Boolean models based on more recent papers, published in 2001 and 2004 [22], [23], shown in Figure 2. The 2004 paper has more hazards than the 2001 paper, and fixing the hazards in the 2004 paper results in a model that is very similar to that from the 2001 paper. The first hazard from the 2004 model is that SK can degrade before Cdc2 is produced. This allows Ste9 and Rum1 to reactivate and prevent production of Cdc2, halting the cell cycle. This hazard can be eliminated by replacing SK self-inhibition with inhibition of SK by Cdc2, as hypothesized in in the 2001 paper.

Another hazard occurs when Slp1 is 1 and Cdc2 is 1. Then either Cdc2 can transition to 0 or Cdc2 can degrade SK to 0. If Cdc2 changes first, SK remains at 1 and the cell cycle arrests. The hazard can be removed be reducing the weight of the inhibitory edge from Slp1 to Cdc2 and replacing constitutive Ste9 production with an activating edge from Slp1 to Ste9 [22].

Finally, one hazard occurs in both models. When Slp1 inhibits Cdc2, causing it to decrease from 2 to 1, Cdc2 is ready to decrease again to 0. However, Slp1 is also enabled to return to 0 because of self-inhibition. If Slp1 transitions first, Cdc2 can again transition to 2, leading to a deviation from the cell cycle. Interestingly, Slp1 and Ste9 are homologous with Cdh1 and Cdc20 in budding yeast, so this is essentially the same hazard that was detected in several budding yeast models above. We could fix the hazard by making the same change that was made in the budding yeast model: replace the self-inhibition of Slp1 by inhibition of Slp1 by Ste9. In budding yeast, there was some evidence in the literature for interaction. Furthermore, similar interaction has been noted in the Xenopus cell cycle, but no evidence of such an interaction has been noted in fission yeast [24]. In general, this hazard can be resolved by ensuring that Slp1 does not return to 0 until Ste9 has transitioned to 1. An alternative revision with this effect would be to replace self-inhibition of Slp1 with inhibition from Wee1 or Rum1. We could find no evidence for or against this possibility in the literature.

### Evolutionary Pressure for Timing Robustness

We tested *in silico* the hypothesis that timing robustness is not an accidental property, but results from natural selection, by introducing random mutations. The models were mutated by deleting, adding, or re-weighting inputs. The mutant networks were checked under the *synchronous* update rule using NuSMV, and those whose cell cycles failed were discarded. The remaining mutants (called “viable mutants”) were further checked for speed independence using NuSMV under the *asynchronous* update rule.

The fraction of viable mutants that are speed-independent declines rapidly as mutations accumulate, as shown in Table 1. From this experiment, it appears that speed-independence is a fragile property that is easily destroyed by random mutations even in cells that are otherwise viable. It is therefore reasonable to conclude that the property is maintained by selection pressure.

**Table 1:** In silico mutation results.

Mutation distance from model	Fraction of viable mutants that are speed-independent in budding yeast	Fraction of viable mutants that are speed-independent in fission yeast
1	0.304	0.202
2	0.216	0.102
3	0.174	0.084
4	0.094	0.056
5	0.062	0.066
6	0.058	0.044

From one to six random mutations were simulated in each model 500 times. Mutants were considered viable if they correctly completed their cell cycles when analyzed with a synchronous update rule. However, mutations tended to reduce timing robustness even in the viable mutants, indicating that timing robustness is maintained by selection.

### Other Notions of Robustness

Robustness, the ability of a system to maintain a function in the presence of perturbations, has been an important theme in systems biology for many years [25]. Different classes of perturbations yield different notions of robustness.

One perturbation is a change of the system state. This leads to one of the notions of robustness explored by the paper by Li, et al. [14]. In that paper, robustness is measured by the size of the “basin of attraction” of G1 (i.e., the set of states which, if the system is started in them, cause it to progress to G1). This notion of robustness is unrelated to timing robustness, and difficult to transfer to asynchronous systems. The definition of the basin of attraction in Boolean systems depends on a state determining a trajectory to a unique attractor. In an asynchronous system, there are usually many trajectories out of any state that visit various attractors.

Another type of robustness is robustness to mutations. One question that has been examined is the relationship between robustness to mutations and evolvability [25]–[27]. Interestingly, our simulation of mutations produces the seemingly opposing result that *timing robustness* is not itself robust to mutations – from which we conclude that it is a property that must be actively maintained by evolution. However, this observation does not necessarily imply that speed independent systems are not robust to mutations. First, timing robustness probably should not be considered a “function,” since a reduction in timing robustness may only change the phenotype of the organism with a small probability. More importantly, timing robustness may also imply mutation robustness because timing robustness depends only on the “wiring diagram” and the logical combinations of input signals at each node. So, a timing-robust organism may freely vary reaction rates without compromising function, allowing evolution to fine-tune and optimize functions smoothly. For example, reaction rates could be freely varied to maximize the speed of the cell cycle or to maximize energy efficiency while preserving the correct function of the cell cycle.

### Are Real Cells Speed-Independent?

There are reasons to suspect that real cells are not quite as timing-robust as the models here seem to suggest. Speed independence may have a cost in time and energy, making it disadvantageous when reactions operate on such different time scales that hazards are unlikely. We conjecture that the observed speed-independence results in part from the simplified representations of the cell cycle models analyzed here, and that more detailed cell cycle models would probably exhibit strong timing robustness while not being completely speed-independent. A reasonable less conservative model would allow for qualitatively different delays [5], [6], [9]. A less conservative model would also allow the possibility that the cell cycle would be fully or partially intact when genes are knocked out, a property of actual cells [18]. It will be possible to answer more of the many open questions about timing robustness as additional details about the regulation of the cell cycle and other cellular processes are discovered.

## Methods

Many of the Boolean networks from the literature are *threshold networks* [11]. Each node can have a 0 or 1 value, and the value of a node when it is next updated is one if the sum of positive inputs minus the of negative inputs exceeds a constant threshold for that node. In analyzing and modifying these models, we found it to be necessary to extend threshold networks to handle more than two values.

Nodes have 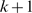 levels of activation, 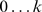, where 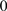 represents a functionally inactive level and 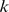 represents maximum activity. Three levels were sufficient for all of the networks appearing here. Each node has a specified threshold between each successive activation level, and each node input has an associated real-valued weight representing the strength of the reaction on the node.

Formally, a Boolean network is defined to have a set of 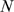 nodes associated with proteins. Each node has a set of inputs, which are other nodes. Each input also has an *input weight*, 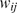. In the networks here, only three magnitudes of weights are required: 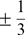, 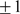, and 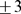. Inputs with positive weights are *activating* and inputs with negative weights are *inhibiting*. Each node 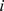 has thresholds 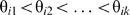. The weighted sum of the inputs to a node are compared with these thresholds to determine whether the node level is enabled to increase or decrease. In the networks here, 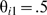 and 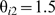 for all nodes.

The network can be depicted as a directed graph. Nodes are labeled circles, and lines with arrowheads (for activating inputs) or flat ends (for inhibiting inputs) represent the inputs to nodes. The thickness of the line represents the weight on that input.

The dynamics of a network are represented as a *Kripke structure*, which is a directed graph of *states*, where each state consists of an assignment of a value of 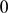 to 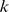 to each node in the network. The Kripke structure has edges from each state to one or more *successor states*; these edges represent possible ways that the states can change over time. The Kripke structure is *deterministic* if each state has exactly one successor. If one or more states have multiple successors, the Kripke structure is *nondeterministic*. The Kripke structure also has a designated *initial state*, which represents a state in which the network can start. The Kripke structure is completely defined by the network and an initial state.

Every possible assignment of values to nodes is a state in the Kripke structure. To define the successors of a state 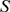, we first define the *next value*, 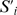, for a node 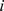 (intuitively the next value is a value that the node will have when next updated, if nothing else changes). 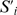 depends on how the weighted sum of inputs to a node compares with thresholds for the node.

Then, the next value can be defined for each node, given a state 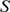, as:
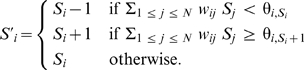
Once the next value of each node is defined, the set of successor states depends on what *update rule* is used. The *synchronous update rule* that was used in much past work defines a single successor of each state 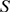 in which each node is given its next value. Intuitively, every node is updated as soon as it can be, and all such nodes are updated simultaneously. Using the synchronous update rule results in a deterministic Kripke structure.

The *asynchronous update rule* specifies that each successor state to 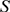 has at most one node updated to its next value (it is always possible that no nodes are updated, in other words, 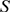 is always a successor of itself). Intuitively, a particular node is chosen arbitrarily for updating. This represents a model with no timing constraints. When , node 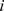 is said to be *enabled*. Using the asynchronous update rule, any node that is enabled may change after an arbitrary delay (but only one such node may change at a time). Any state that has at least one enabled node has multiple successor states, so the Kripke structure is nondeterministic.

### NuSMV

Network behavior (under the synchronous and asynchronous update rules) was analyzed using a symbolic model checking tool, NuSMV, to test the robustness of the cell cycle model [8]. NuSMV explores the paths of states starting from the initial state to check a specific property of interest.

The theory and algorithms in NuSMV are the results of several decades of research published in many books and papers, so we cannot explain them in depth. In brief, NuSMV is able to check dynamic properties of finite-state systems for infinitely many possible variations in inputs or internal non-deterministic choices by exhaustively searching the finite states of the system for individual states or loops that violate the property. NuSMV has several alternative implementations for searching the state of states. We used the implementation based on Boolean decision diagrams (BDDs), a data structure that can be used to explore large state sets relatively quickly (however, the models of the cell cycle only have a few thousand states, so any reasonable implementation of model checking should work well on them).

NuSMV has an input language, similar to a programming language, for describing the system to be checked. Descriptions in the language consist of a set of state variables ranging over discrete sets of values, along with rules that update those variables based on the previous state. These rules can be non-deterministic, meaning that state variables can be updated to several alternative values, in which case NuSMV will check the property for all of those possibilities. The NuSMV input language is naturally synchronous: time is divided into steps, and all variables are updated on each step. However, asynchronous updating can be encoded in NuSMV by exploiting non-determinism. Each reaction in SMV was coded as a separate rule updating its reactants and products. To implement the asynchronous update rule, an additional control variable was added to the rule, along with logic that only allows the rule to update if the control variable is set to a particular value that triggers that rule. At each time step, a value is non-deterministically chosen for the control variable, so at most one rule can fire at a time. To implement the synchronous update rule, the control variables are omitted from the rules so that all of them update simultaneously.

The property is written in NuSMV's logical specification language, CTL. It requires that the cell *inevitably* reach G1 for all of the sequences of states that occur with the chosen update rule. This property is violated if the cell cycle halts in some other state or continues without reaching G1. For the budding yeast models, G1 is considered to be the state where Sic1 and Cdh1 are 1 and all other variables are 0, and the initial state is the same except Cln3 is also 1. For fission yeast, G1 is taken to be the state where Wee1, Ste9 and Rum1 are 1 and all other variables are 0, and the initial state is the same except SK is also 1. We have also checked the more stringent (and complex) properties that the cell progresses through the correct sequence of stages as it progresses to G1. The models are not detailed enough to specify many intermediate states, but, in budding yeast, Clb5 is associated with DNA replication and Clb2 is associated with mitosis. In the final budding yeast model, as expected, a property requiring that the Clb5 go to 1 before Clb2 goes to 1, before finally returning to G1, holds for all possibly reaction delays. The final fission yeast model is even smaller than the budding yeast model, but we can check that Cdc2, the main driver protein causes both replication and mitosis, reaches its highest level.

The source descriptions of the models and a translator to generate NuSMV descriptions from them are available at http://verify.stanford.edu/TimingRobustnessInYeast.html. NuSMV is a widely-used open source system that can easily found by searching the Web.

### Mutation Simulation

To test whether there is selection pressure on the cells to be speed-independent, the speed-independent models for both budding yeast and fission yeast were mutated *in silico*. The mutations were chosen by first choosing, with probability 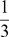, whether to add an input, delete an input, or re-weight an input. If the decision was to add an input, nodes 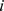 and 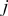 were chosen with equal probability and node 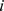 was added as an input to 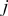 with a non-zero weight chosen with equal probability from 
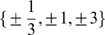
. If the decision was to delete in input, a pair of nodes 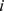 and 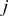 were chosen with equal probability, where 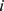 was an input to 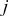, and 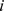 was deleted from the set of inputs of 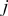. If re-weighting was chosen, an input was selected at random and the weight was changed with equal probability from 
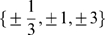
, excluding the current input weight.

Mutations were performed in a series of steps. At each step, mutants were checked with NuSMV, using the CTL properties above, but with a *synchronous* update rule. Mutants that failed this test were considered “non-viable” and discarded. The viable mutants were further checked using NuSMV and the *asynchronous* update rule to discover the proportion that were speed-independent. 500 viable mutants were generated at each mutation step, from 1 to 6 steps.
